# Three-dimensional porous carbon composites containing high sulfur nanoparticle content for high-performance lithium–sulfur batteries

**DOI:** 10.1038/ncomms10601

**Published:** 2016-02-01

**Authors:** Guoxing Li, Jinhua Sun, Wenpeng Hou, Shidong Jiang, Yong Huang, Jianxin Geng

**Affiliations:** 1 Technical Institute of Physics and Chemistry, Chinese Academy of Sciences, 29 Zhongguancun East Road, Haidian District, Beijing 100190, China

## Abstract

Sulfur is a promising cathode material for lithium–sulfur batteries because of its high theoretical capacity (1,675 mA h g^−1^); however, its low electrical conductivity and the instability of sulfur-based electrodes limit its practical application. Here we report a facile *in situ* method for preparing three-dimensional porous graphitic carbon composites containing sulfur nanoparticles (3D S@PGC). With this strategy, the sulfur content of the composites can be tuned to a high level (up to 90 wt%). Because of the high sulfur content, the nanoscale distribution of the sulfur particles, and the covalent bonding between the sulfur and the PGC, the developed 3D S@PGC cathodes exhibit excellent performance, with a high sulfur utilization, high specific capacity (1,382, 1,242 and 1,115 mA h g^−1^ at 0.5, 1 and 2 C, respectively), long cycling life (small capacity decay of 0.039% per cycle over 1,000 cycles at 2 C) and excellent rate capability at a high charge/discharge current.

## Abstract (web summary)

Lithium–sulfur batteries have high theoretical capacities but their performance is limited by poor conductivity and low stability. Here, the authors fabricate three-dimensional porous graphitic carbon composites containing sulfur nanoparticles and probe the effect of sulfur content on battery performance.

## Graphical abstract

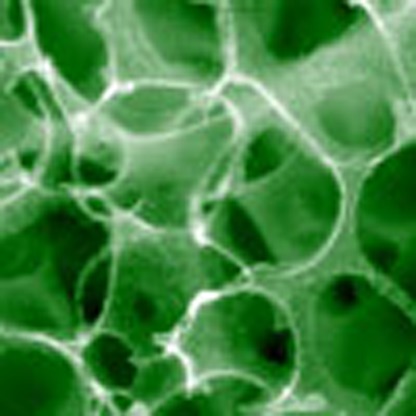

Lithium–sulfur (Li–S) batteries have recently attracted great interest as promising electrochemical devices for energy conversion and storage applications because of the abundance, low cost, environmental friendliness and high theoretical capacity (1,675 mA h g^−1^) of sulfur^1^,^2^,^3^,^4^. Despite these advantages, the practical application of Li–S batteries is still handicapped by the following problems: (1) the low electrical conductivities of sulfur (5 × 10^−30^ S cm^−1^ at 25 °C), intermediate polysulphides and Li_2_S; (2) the dissolution of lithium polysulphides, which results in a shuttling effect and in the deposition of insoluble lithium sulfide on the anode in each of the charge/discharge cycles and eventually the complete loss of capacity of the sulfur cathode; and (3) severe volume changes in the active electrode materials during the lithiation/delithiation processes^1^,^4^,^5^,^6^,^7^, resulting in the pulverization of the electrode materials.

To overcome these problems, various carbon materials, including graphene^8^,^9^,^10^,^11^,^12^,^13^,^14^,^15^, carbon nanotubes^16^,^17^, porous carbon^18^,^19^,^20^,^21^,^22^,^23^,^24^,^25^,^26^ and carbon nanofibres^27^,^28^,^29^, have been tested in recent years as supporting materials for sulfur cathodes to improve the electrochemical performance of Li–S batteries. Carbon frameworks improve the electrical conductivity of sulfur cathodes and trap soluble polysulphides during cycling. In addition, yolk–shell structures such as a sulfur–TiO_2_ yolk–shell^30^ and a sulfur–polyaniline yolk–shell^31^ have been developed to address the large volume changes of sulfur during the lithiation/delithiation processes. Recently, Choi *et al.* fabricated a polydopamine-coated S/C composite cathode with a high sulfur loading, which exhibited a high areal capacity (9 mA h cm^−2^) (ref. 32). In a pioneering work, Pyun and co-workers prepared sulfur-containing polymers that exhibited high electrochemical activity and suitability as cathode materials for Li–S batteries^33^,^34^. However, despite these research efforts, no strategy has satisfactorily solved the aforementioned problems. Most seriously, the long-cycle stability under high charge/discharge rates remains a major challenge for sulfur-based cathodes, especially for composites with relatively high sulfur content. To date, conventional methods for the preparation of carbon–sulfur composites^8^,^16^,^19^,^20^,^21^,^35^, in which carbon materials are impregnated with sulfur by diffusion after the carbon structures are prepared, still face challenges. These problems include the complexity of multistep operations, the low sulfur content of the composites and the out-diffusion of lithium polysulphide into the electrolyte during the charge/discharge cycles owing to the diffusion process used for the incorporation of sulfur into the carbon materials. Furthermore, certain unresolvable trade-offs have been found in previous studies. For example, a relatively high sulfur content in the sulfur/carbon hybrid structures is always accompanied by larger sulfur particles^11^,^30^,^36^, which severely reduces the rate of sulfur utilization because of the long diffusion path for electrons and lithium ions^3^. Although a very high specific capacity (>1,000 mA h g^−1^) can be obtained with an electrode that has a low sulfur content^37^,^38^,^39^,^40^, the low sulfur content greatly reduces the overall volumetric capacity and energy density of the cathode. Therefore, it is crucial to design high-sulfur-content composites for use as cathode materials in Li–S batteries, in which the composite cathodes maintain a high sulfur utilization rate, a high specific capacitance, a long cycling life and good rate capability. This may be achievable by controlling the existing state and distribution of sulfur in the hybrid structures.

Herein, we report a facile and scalable strategy for the *in situ* synthesis of sulfur nanoparticles in three-dimensional (3D) porous graphitic carbon (PGC) (designated 3D S@PGC) with a tuneable sulfur content and demonstrate the utility of the 3D S@PGC as a cathode material for Li–S batteries. Compared with the conventional methods for the preparation of carbon–sulfur composites^8^,^16^,^19^,^20^,^21^,^35^, our strategy facilitates access to composites that have the advantages of a high sulfur content (up to 90 wt%) and nanoscale distribution of the sulfur particles, as well as covalent bonding between the sulfur nanoparticles and the PGC network, which ensures the efficient utilization of the loaded sulfur. The sulfur content of the composite can be readily tuned by changing the Na_2_S/glucose ratio. Because of the C–S bonds, unique interconnected hierarchical porous structures, high sulfur content and nanoscale sulfur particles, our 3D S@PGC (90% S) composite exhibits significantly improved electrochemical performance as a cathode material for Li–S batteries. In particular, this material has a high specific capacity (1,382, 1,242 and 1,115 mA h g^−1^ at 0.5, 1 and 2 C, respectively), long cycling life (small capacity decay of 0.039% per cycle over 1,000 cycles at 2 C), and excellent rate capability at a high charge/discharge current.

## Results

### Synthesis of the 3D S@PGC composites

**Figure 1:**
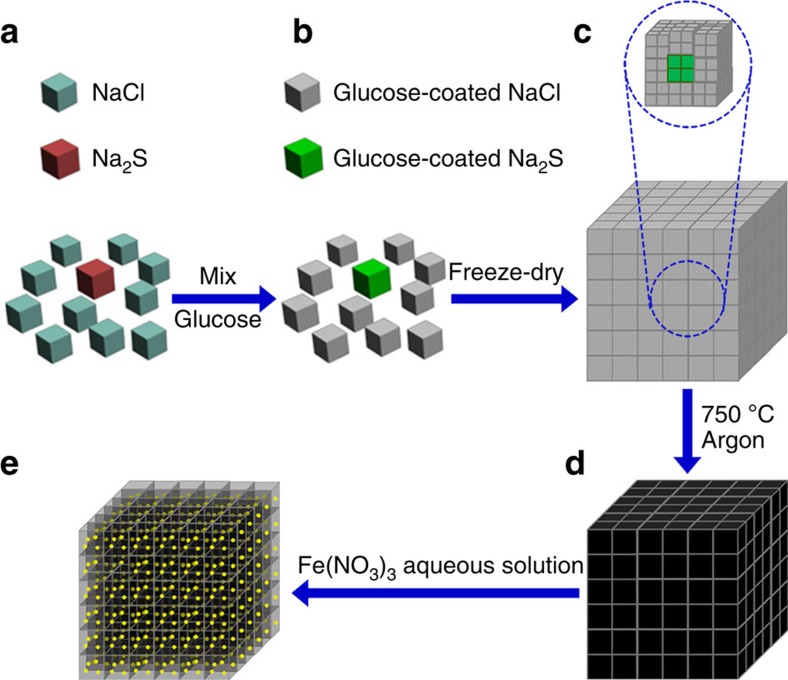
Schematic illustration of an *in situ* strategy for the preparation of 3D S@PGC composites. (**a**) NaCl and Na_2_S crystals. (**b**) Glucose-coated NaCl and Na_2_S crystals. (**c**) Self-stacking of the glucose-coated NaCl and Na_2_S crystals (3D NaCl–Na_2_S@glucose), with Na_2_S crystals surrounded by NaCl crystals. (**d**) Self-stacking of GC-coated NaCl and Na_2_S crystals (3D NaCl–Na_2_S@GC). (**e**) 3D S@PGC composite formed through the simultaneous dissolution of the NaCl crystals and oxidation of the Na_2_S with Fe(NO_3_)_3_.

Figure 1 displays the scheme for the preparation of the 3D S@PGC composites. The self-stacking of water-soluble NaCl and Na_2_S crystals was utilized to form a hard template for the 3D PGC network. Na_2_S, which reacts with Fe(NO_3_)_3_ to form sulfur, was used as the sulfur precursor. The reaction is described by the equation Na_2_S+2Fe(NO_3_)_3_→2Fe(NO_3_)_2_+2NaNO_3_+S. NaCl, Na_2_S and glucose were first dissolved in deionized (DI) water to obtain a homogeneous solution, which was subsequently subjected to freeze-drying. During the freezing process, the sizes of the NaCl and Na_2_S crystals were restricted, and the crystals were uniformly coated with an ultrathin glucose film. The NaCl crystals surrounded the Na_2_S crystals because of the high NaCl/Na_2_S molar ratio (10:1). The hybrid structure is hereafter referred to as 3D NaCl–Na_2_S@glucose. Upon heating at a high temperature (750 °C) in an argon atmosphere, the glucose underwent carbonization to form graphitic carbon (GC) with micro- and mesopores^41^,^42^, thus leading to 3D NaCl–Na_2_S@GC. Finally, the immersion of 3D NaCl–Na_2_S@GC in an aqueous Fe(NO_3_)_3_ solution dissolved the NaCl template, leaving macropores in the composite. Concurrently, sulfur nanoparticles formed *in situ* and deposited on the walls of the macropores through the oxidation of Na_2_S by Fe(NO_3_)_3_. During this process, the Na_2_S that was dissolved in the aqueous Fe(NO_3_)_3_ solution in the 3D PGC macropores diffused into the micro- and mesopores because of capillarity, allowing the micro- and mesopores to fill with sulfur. Hence, the sulfur nanoparticles were uniformly distributed in the 3D PGC through an *in situ* chemical deposition process. According to previous reports^18^,^19^, the PGC content, 3D porous architecture and sulfur content are crucial parameters that determine the properties of batteries such as the electrical conductivity of the electrode, electrolyte transport in the electrode and specific capacity. In our research, NaCl and Na_2_S crystals were used as a template to fabricate the 3D porous architecture, and the sulfur content of the composites could be readily tuned by changing the Na_2_S/glucose ratio. A 3D S@PGC composite with a sulfur content of up to 90 wt% [3D S@PGC (90% S)] was obtained by optimizing the Na_2_S/glucose ratio (1:0.4). Therefore, our method has advantages over conventional methods^8^,^16^,^19^,^20^,^21^,^35^ because the composition and structure of the composites can be easily controlled.

### Structure and morphology of the 3D S@PGC composites

**Figure 2:**
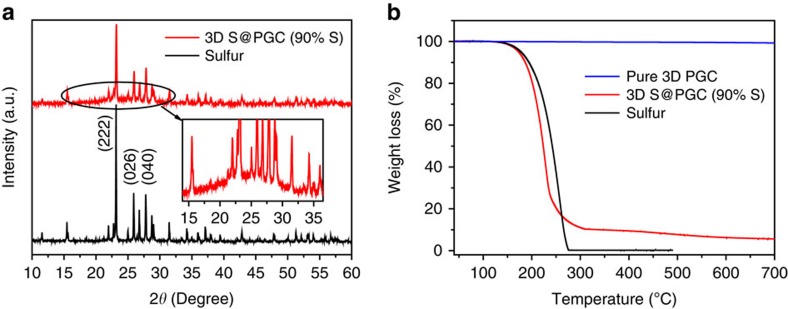
Structural analysis of the 3D S@PGC (90% S) composite. (**a**) X-ray diffraction patterns of sulfur (bottom trace) and 3D S@PGC (90% S) composite (top trace), which confirm the crystalline feature of the sulfur nanoparticles in the 3D PGC. (**b**) TGA curves of sulfur, pure 3D PGC and the 3D S@PGC (90% S) composite. The 3D PGC apparently did not undergo weight loss up to 700 °C; the calculated sulfur content of the composite is thus ∼90 wt%.

The crystallographic structure of the 3D S@PGC (90% S) composite was first analysed using X-ray diffraction (Fig. 2a). The X-ray diffraction pattern of elemental sulfur has three prominent Bragg reflections at 23.1, 25.9 and 27.8° (bottom trace), which can be indexed as the (222), (026) and (040) planes of the *fddd* orthorhombic structure^43^. As expected, the 3D S@PGC (90% S) composite produced a similar pattern, confirming the crystalline feature of the sulfur nanoparticles in the 3D PGC. The broad reflection peak, which can be clearly identified in the range of 15–35° (inset of Fig. 2a and Supplementary Fig. 1), is attributed to PGC. The peak for PGC in the 3D S@PGC (90% S) is weak because of the relatively low amount of PGC in the composite. A thermogravimetric analysis (TGA) indicated that the sulfur content of the 3D S@PGC (90% S) composite is ∼90 wt% (Fig. 2b).

**Figure 3:**
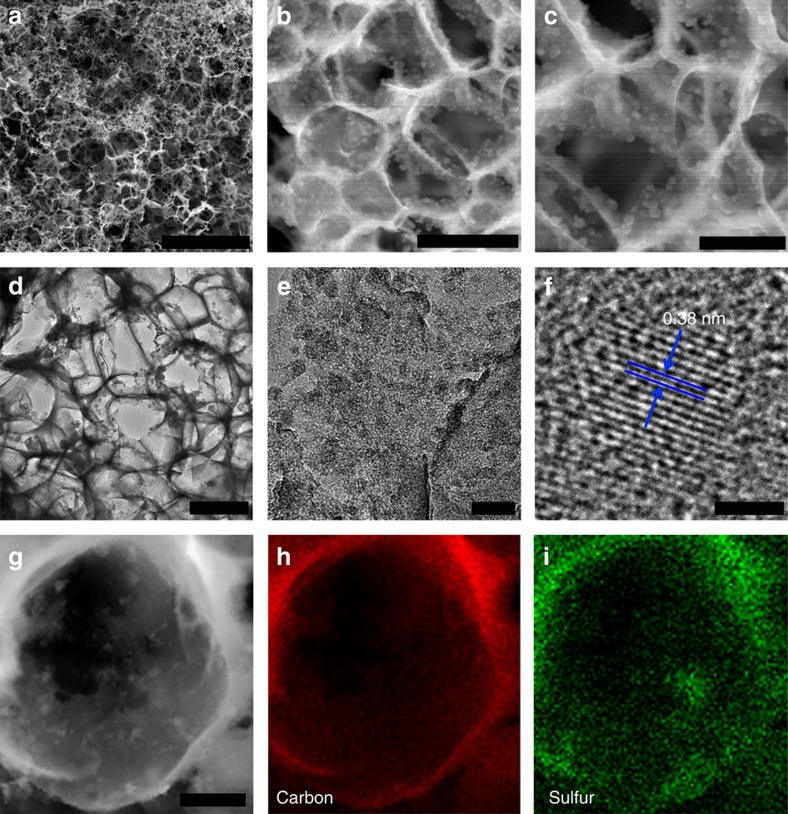
Morphology of the 3D S@PGC (90% S) composite. (**a**–**c**) SEM images and (**d**,**e**) TEM images of the 3D S@PGC (90% S) composite at different magnifications. (**f**) HRTEM image of a sulfur nanoparticle in the composite. (**g**) TEM image of the 3D S@PGC (90% S) composite. (**h**,**i**) EDS elemental maps of (**h**) carbon and (**i**) sulfur, which were collected from the entire area shown in **g**. Scale bars in **a**,**b** and **c**: 20; 1; and 0.5 μm. Scale bars in **d**,**e**,**f** and **g**: 500; 50; 2; and 200 nm. The SEM and TEM images indicate that the composite possesses a 3D network consisting of interconnected submicron-sized macropores. From the SEM images, the sulfur nanoparticles anchored to the walls of the PGC network were calculated to have a size distribution of 18–54 nm. The EDS results indicate that the sulfur is uniformly distributed in the composite. HRTEM, high-resolution TEM.

The morphology of the 3D S@PGC (90% S) composite was investigated via electron microscopy. Figure 3a displays an overview of the composite, with its honeycomb-like porous surface, obtained via scanning electron microscopy (SEM). At higher magnification (Fig. 3b), the unique network of the composite, which is composed of interconnected submicron-sized macropores, can be seen. With a further increase in magnification (Fig. 3c), sulfur nanoparticles with sizes ranging from 18 to 54 nm, homogeneously and densely anchored onto the walls of the PGC network, can be observed (Supplementary Fig. 2). A comparison of these morphologies with those of the 3D NaCl–Na_2_S@GC and the pure 3D PGC framework (Supplementary Figs 3 and 4) led us to conclude that the 3D self-stacking of the NaCl and Na_2_S crystals had been well preserved after carbonization. Therefore, the SEM data support the formation mechanism of the 3D S@PGC composites (Fig. 1). An image obtained via transmission electron microscopy (TEM) reveals that the pores overlap each other and form a continuous 3D network with ultrathin GC walls (Fig. 3d). In good agreement with the SEM data, the ultrathin carbon walls are uniformly covered with sulfur nanoparticles (Fig. 3e). The composites that were subjected to an elongated period of sonication resulted in similar TEM images (Supplementary Fig. 5). The TEM results indicate that the attachment of the sulfur nanoparticles to the walls of the PGC network was robust because the materials used for TEM observation were subjected to long periods of sonication during the sample preparation. Moreover, the 3D porous structure remained intact, demonstrating the high mechanical flexibility of the 3D S@PGC composites. A high-resolution TEM image reveals the crystalline feature of the sulfur nanoparticles (Fig. 3f). Clear lattice fringes with an interlayer spacing of 0.38 nm corresponding to the (222) planes are readily observable, as the (222) Bragg reflection has the greatest intensity in the X-ray diffraction pattern (Fig. 2a). Energy-dispersive X-ray spectroscopy (EDS) elemental mapping confirmed the presence of carbon and sulfur in the 3D S@PGC (90% S) composite (Fig. 3g–i), as well as the homogenous distribution of sulfur nanoparticles in the PGC framework (Fig. 3i, sulfur mapping). A nanosized distribution is extremely important for the application of the sulfur particles as a cathode material for Li–S batteries, as the utilization rate of sulfur is higher for smaller sulfur particles because of the short diffusion path of the electrons and lithium ions^3^. Compared with previously reported hybrid structures that have a comparably high sulfur content (∼70 wt%) (refs 10, 11, 30), the sulfur particles in the 3D S@PGC (90% S) were much smaller and had a much more uniform distribution. These features can be attributed to the simultaneous formation of the porous structures and the *in situ* deposition of sulfur nanoparticles through the oxidation of Na_2_S.

**Figure 4:**
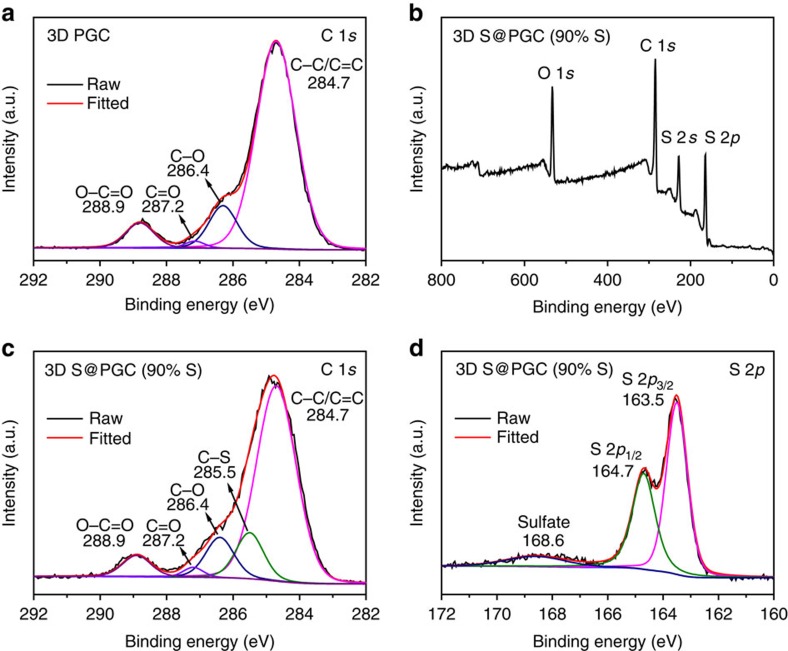
XPS results for pure 3D PGC and the 3D S@PGC (90% S) composite. (**a**) C 1*s* XPS spectrum of pure PGC. (**b**) XPS survey spectrum of the 3D S@PGC (90% S) composite. (**c**) C 1*s* and (**d**) S 2*p* XPS spectra of the 3D S@PGC (90% S) composite. The data indicate the presence of C–S bonds in the 3D S@PGC (90% S) composite.

To further investigate the interactions between sulfur and 3D PGC, X-ray photoelectron spectroscopy (XPS) was performed. For comparison, XPS data were also collected for pure 3D PGC, which was prepared by immersing NaCl–Na_2_S@GC in water to remove NaCl and Na_2_S (Fig. 4a). The C 1*s* XPS spectrum of pure 3D PGC has a major peak at 284.7 eV, corresponding to *sp*^2^ hybridized carbon, as well as three weak peaks at 286.4, 287.2 and 288.9 eV, which can be ascribed to C–O, C=O and O–C=O species, respectively^14^. The survey XPS spectrum of the 3D S@PGC (90% S) composite confirms the presence of sulfur in 3D PGC (Fig. 4b). In contrast to the C 1*s* XPS spectrum of pure 3D PGC, that of the 3D S@PGC (90% S) composite has an additional peak at 285.5 eV, which is ascribed to C–S bonds (Fig. 4c)^14^. This finding reveals the presence of covalent bonding between sulfur and PGC. The S 2*p* XPS peaks, that are characterized by an S 2*p*_3/2_ and 2*p*_1/2_ doublet with an energy separation of 1.2 eV, reconfirm the presence of C–S bonds (Fig. 4d), as the binding energy of the S 2*p*_3/2_ peak (163.5 eV) is lower than that of elemental sulfur (164.0 eV)^14^,^44^. The weak peak at 168.6 eV is due to sulfate species formed by the oxidation of sulfur in air^14^. The presence of C–S bonds is also supported by Fourier transform infrared (FTIR) spectroscopy because the vibration characteristic of C–S bonds was detected at 671 cm^−1^ (Supplementary Fig. 6)^45^. The C–S bonds could be formed through the addition of various reactive intermediates, including free radicals (for example, HS^·^) and radical anions (for example, S^·−^ and S_X_^·−^)^46^, to the unsaturated carbon–carbon double bonds of the PGC as well as through the nucleophilic attack of transient negatively charged polysulphides (for example, S_X_^ 2−^) with residual oxygen-containing functional groups present in the PGC (see Supplementary Note 1). Therefore, the C−S bonds were formed during the oxidation of Na_2_S by Fe(NO_3_)_3_ because free radicals, radical anions and negatively charged polysulphides were the intermediate products of the oxidation reaction^46^.

To reconfirm the presence of C−S bonds, the 3D S@PGC (90% S) composite was subjected to Soxhlet extraction using CS_2_. The TGA curve of the extracted sample revealed a continual weight loss up to 700 °C (Supplementary Fig. 7); such a weight loss could be assigned to the removal of bonded sulfur^47^, and the percentage of bonded sulfur was estimated to be ∼48 wt%. By comparing the sulfur content of the as-prepared 3D S@PGC (90% S) composite to that of the Soxhlet-extracted sample, the bonded and unbonded sulfur content were calculated to be ∼9 and 81 wt% in the 3D S@PGC (90% S) composite (Supplementary Table 1). The X-ray diffraction pattern of the extracted sample did not have any sulfur peaks (Supplementary Fig. 8), which is in line with the formation of C−S bonds^48^. Compared with previously reported physical strategies for confining sulfur^21^,^23^,^25^,^49^,^50^, covalent bonding between sulfur nanoparticles and the PGC framework should effectively prevent the loss of the active materials and stabilize the cycling life of the corresponding Li–S batteries.

**Figure 5:**
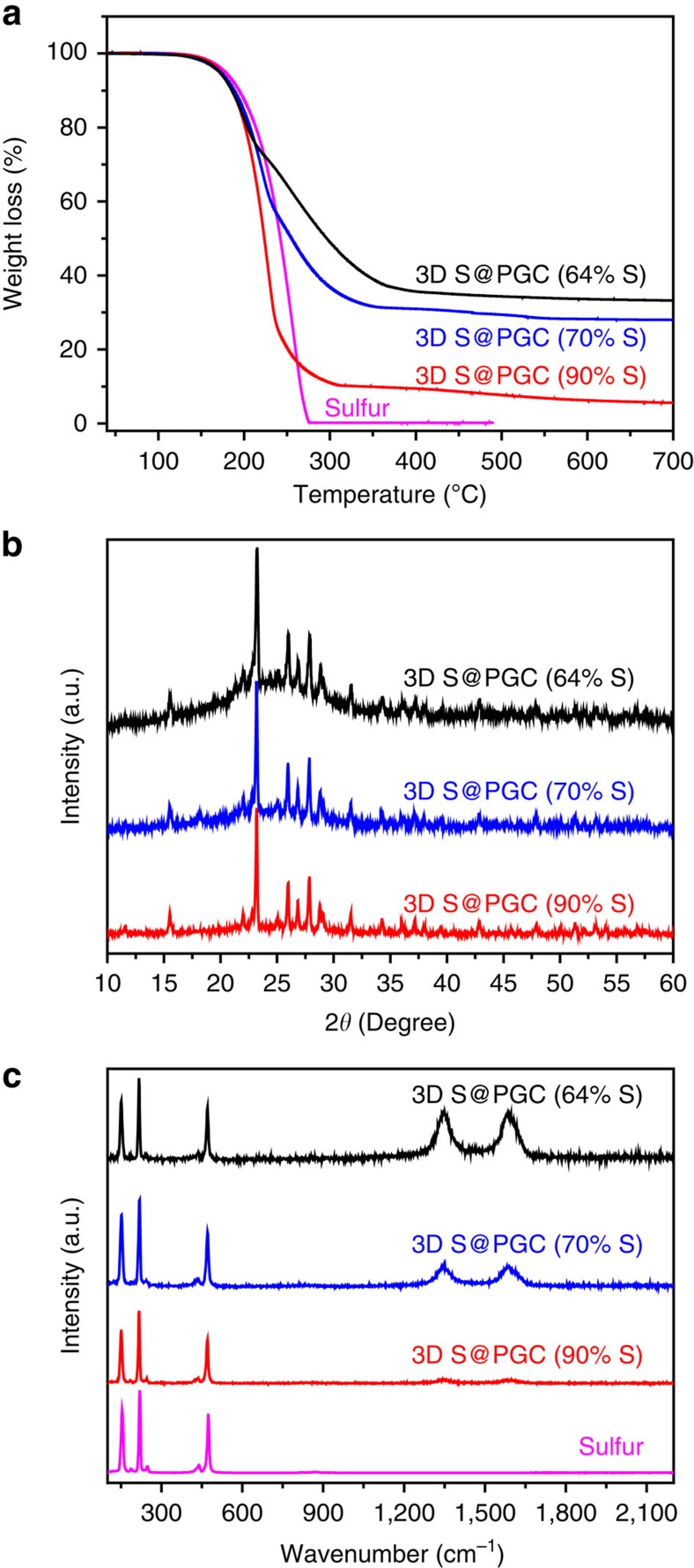
Characterization of 3D S@PGC composites with various sulfur contents. (**a**) TGA curves, (**b**) X-ray diffraction patterns and (**c**) Raman spectra reveal that the sulfur content in the 3D S@PGC composites can be readily tuned by changing the Na_2_S/glucose ratio and that the sulfur nanoparticles in the composites have the same crystalline structures.

The sulfur content of our 3D S@PGC composites is readily tuneable. To tune the amount of sulfur, we used different Na_2_S/glucose ratios (that is, 1:0.5, 1:0.45 and 1:0.4) during the preparation while maintaining a constant Na_2_S content (2.0 g). Figure 5 displays the TGA, X-ray diffraction and Raman spectroscopy data of 3D S@PGC composites prepared with different Na_2_S/glucose ratios. The sulfur content calculated from the TGA curves for the 3D S@PGC composites prepared with the aforementioned ratios was ∼64, 70 and 90 wt%, respectively (Fig. 5a). The 3D S@PGC composites exhibited weight losses at different stages. Compared with pure sulfur, the 3D S@PGC composites exhibited slightly lower starting evaporation temperatures. The evaporation of sulfur in this temperature range was ascribed to the sulfur nanoparticles attached to the walls of the PGC without covalent bonding. This lowering of the evaporation temperature was mainly ascribed to the nanosized distribution of the sulfur particles, which possessed excess surface free energy and facilitated heat transfer because of the large contact area between the sulfur nanoparticles and the 3D PGC (ref. 51). The 3D S@PGC composites also exhibited evaporation temperature ranges that were higher than that of pure sulfur. This phenomenon was caused by the sulfur that filled the micro- and mesopores^52^ and covalently bonded to the wall surfaces of the PGC^47^. The 3D S@PGC (64% S) composite exhibited the most obvious weight loss at a relatively higher temperature because the proportion of sulfur contained within the micro- and mesopores and bonded to the PGC relative to the total amount of sulfur was the highest among the three composites. The X-ray diffraction patterns of the composites contain reflection peaks for both sulfur (23.1°, 25.9° and 27.8°) and GC (broad peak from 15 to 35°) (Fig. 5b and Supplementary Fig. 1), indicating the presence of sulfur in the 3D PGC. With the decrease in the PGC content of the composites, the intensity of the PGC peak became weaker. Collectively, the sulfur content of the 3D S@PGC composites increased with the amount of Na_2_S used in the synthetic procedure (Supplementary Table 1).

The structural features of sulfur and the carbon matrix of the 3D S@PGC composites were further investigated by Raman spectroscopy (Fig. 5c). Elemental sulfur produced characteristic peaks at 471, 216 and 151 cm^−1^. In agreement with the XRD result, the Raman spectra of the 3D S@PGC composites contain characteristic peaks of sulfur. Furthermore, the Raman spectra of the composites present peaks at ∼1,350 and 1,588 cm^−1^, which correspond to the D and G bands of carbon materials^53^. The presence of such bands in the Raman spectra suggests the conversion of glucose into GC. With the decrease in the PGC content of the composites, the D and G bands also became weaker.

The SEM and TEM images (Supplementary Figs 9 and 10) reveal that 3D S@PGC composites with different amounts of sulfur display similar morphologies, indicating the retention of the unique honeycomb-like network in the composites. Although the sizes of the sulfur nanoparticles calculated from the SEM images increased with the sulfur content (that is, 6–24 nm for 3D S@PGC (64% S), 12–35 nm for 3D S@PGC (70% S) and 18–54 nm for 3D S@PGC (90% S); Supplementary Fig. 2 and Supplementary Table 1), the sulfur particle size found in the 3D S@PGC (90% S) composite (18–54 nm) still resulted in high sulfur utilization, as discussed below. The sizes of the sulfur nanoparticles calculated from the Scherrer formula using the (222) Bragg reflection at 23.1° (∼28, 33 and 44 nm for the 3D S@PGC (64% S), 3D S@PGC (70% S) and 3D S@PGC (90% S) composites, respectively) are in agreement with the values obtained from the SEM images. The FTIR spectra and XPS data confirm that C–S bonds also exist in the 3D S@PGC (70% S) and 3D S@PGC (64%) composites (Supplementary Figs 6 and 11). The pore structures of the 3D S@PGC composites were also characterized using N_2_ physisorption measurements, providing insight into the micro- and mesopore structures of the composites (Supplementary Fig. 12 and Supplementary Note 2). All the 3D S@PGC composites had smaller surface areas and pore volumes than the corresponding PGC frameworks obtained by immersing NaCl–Na_2_S@GC in water. The marked decreases in the specific surface area and total pore volume upon the deposition of sulfur nanoparticles indicated that the sulfur occupied the volumes of the pores in the 3D PGC frameworks. A comparison of the pore-size distributions of the 3D S@PGC composites and the corresponding 3D PGC frameworks indicated that the latter contained numerous micro- and mesopores, with sizes ranging from 1.5 to 25 nm, which were filled during formation of the composites. Combining the data from the SEM, TEM and N_2_ physisorption analyses, we can conclude that the sulfur situates both in the micro-/mesopores and on the walls of the macropores of the 3D PGC.

### The 3D S@PGC composites for Li–S battery cathode materials

**Figure 6:**
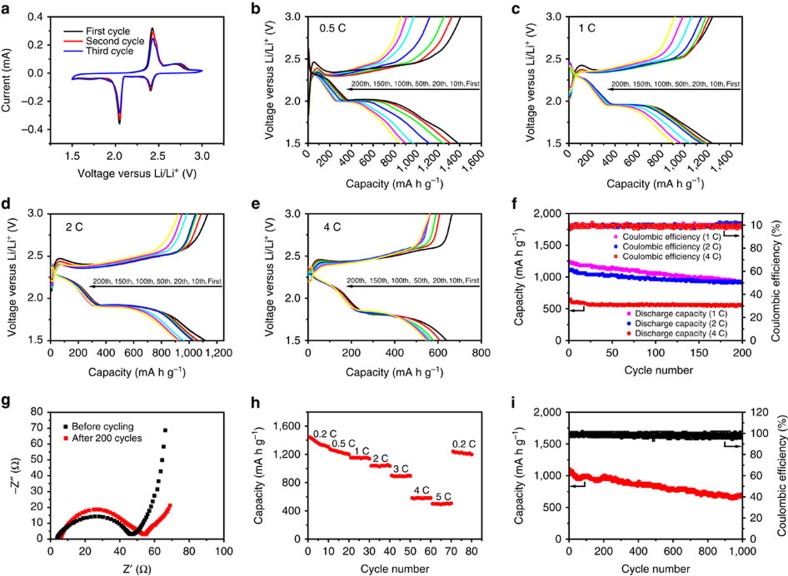
Electrochemical performance of the 3D S@PGC (90% S) composite as a cathode material for Li–S batteries. (**a**) CV profiles of the 3D S@PGC (90% S) composite cathode. (**b**–**e**) Charge/discharge curves of the cathode at charge/discharge rates of (**b**) 0.5 C, (**c**) 1 C, (**d**) 2 C and (**e**) 4 C. (**f**) Cycling performance of the cathode at charge/discharge rates of 1, 2 and 4 C. (**g**) EIS curves of the cathode before and after 200 cycles at 2 C. (**h**) Rate performance of the cathode. (**i**) Cycling performance of the cathode over 1,000 cycles at a charge/discharge rate of 2 C. The data indicate that the 3D S@PGC (90% S) composite displays excellent electrochemical performance, in particular, a high sulfur utilization, high specific capacity (1,115 mA h g^−1^ at 2 C), long cycling life (small capacity decay of 0.039% per cycle over 1,000 cycles at 2 C), and excellent rate capability at a high charge/discharge current. CV, cyclic voltammetry. EIS, electrochemical impedance spectroscopy.

Coin cells with Li foil as an anode were fabricated to evaluate the electrochemical performance of the 3D S@PGC (90% S) composite as a cathode material. Profiles obtained by cyclic voltammetry conducted at a scan rate of 0.1 mV s^−1^ are presented in Fig. 6a. Well-defined reduction peaks were observed at 2.37 and 2.04 V, indicating that the sulfur was reduced in two stages. According to a reported mechanism^15^, the peak at 2.37 V corresponds to the reduction of elemental sulfur to lithium polysulphides (Li_2_S_*n*_, 4≤*n* ≤8), and the peak at 2.04 V corresponds to the further reduction of the lithium polysulphides to Li_2_S_2_ and, eventually, to Li_2_S. The weak peak at ∼1.7 V is assigned to the reduction of LiNO_3_. Likewise, the oxidation of the cathode also proceeded through two stages: the conversion of Li_2_S_2_/Li_2_S to Li_2_S_*n*_ (*n*>2), associated with the oxidation peak at 2.43 V, and the final formation of elemental sulfur, corresponding to the oxidation peak at 2.47 V. The galvanostatic charge/discharge behaviour of the 3D S@PGC (90% S) cathode was studied at charge/discharge rates of 0.5, 1, 2 and 4 C within a potential window of 1.5–3.0 V versus Li^+^/Li (Fig. 6b–e). Consistent with the two reduction peaks at 2.37 and 2.04 V in the cathodic sweep, two plateaus were observed at ∼2.3 and 2.0 V in the discharge process at 0.5 C, corresponding to the two-stage reduction of elemental sulfur to lithium polysulphides (Li_2_S_4–8_) and then to Li_2_S_2_/Li_2_S, respectively. Discharge curves obtained at higher currents (1, 2 and 4 C) also clearly contain the two plateaus, indicating that the electrochemical reactions at higher charge/discharge rates follow processes similar to those occurring at lower rates. However, the non-conductive nature of Li_2_S_2_ and Li_2_S subjects the conversion of Li_2_S_4–8_ to Li_2_S_2_/Li_2_S to higher polarization at higher charge/discharge rates, that is, decreased voltage at the second plateau^54^. As previously noted^16^,^47^,^55^,^56^, the charge/discharge profiles can be sloppy at the ends. This phenomenon could be ascribed to the presence of C–S bonds^16^,^47^ and the strong absorption of sulfur in the micro- and mesopores of the 3D PGC^55^,^56^, as well as the irreversible reduction of LiNO_3_ (refs 57, 58).

The cycling performance of the 3D S@PGC (90% S) composite cathode was first studied at 0.5 C (Supplementary Fig. 13). The material achieved an initial discharge capacity as high as 1,382 mA h g^−1^, which corresponds to 82.5% sulfur utilization based on the theoretical value of sulfur (1,675 mA h g^−1^). After 200 cycles, the calculated capacity retention was 62%. The high utilization rate of sulfur is because of the hierarchical porous structures and the nanosized sulfur particles distributed on the GC walls. The macropores highly favour the rapid access to the electrode interior by the electrolyte, while the nanosized sulfur particles and ultrathin GC walls with micro- and mesopores facilitate the efficient transport of ions into the deeper portions of the sulfur nanoparticles because of the short pathway^18^,^23^,^41^. Figure 6f displays the cycling performance of the 3D S@PGC (90% S) composite cathodes at charge/discharge rates of 1, 2 and 4 C. The initial discharge capacity was 1,242 mA h g^−1^ at 1 C. After 200 cycles, a capacity of 917 mA h g^−1^ remained, corresponding to a capacity retention of 74%. When the charge/discharge rate was raised to 2 C, the initial capacity obtained (1,115 mA h g^−1^) became lower than the corresponding value at 1 C, but the cycling stability improved. A capacity of 920 mA h g^−1^ corresponding to a capacity retention of 83% was obtained after 200 cycles at 2 C. When the charge/discharge rate was further elevated to 4 C, the measured initial capacity was 638 mA h g^−1^. After 200 cycles, the capacity remained at 548 mA h g^−1^, which corresponds to a capacity retention of 86%. The capacities of the composite obtained at current densities of 0.5, 1 and 2 C (that is, 1,382, 1,242 and 1,115 mA h g^−1^ at 0.5, 1 and 2 C, respectively) are much higher than those of previously reported sulfur–carbon composites (430–1,100 mA h g^−1^ at 0.5 C, 400–980 mA h g^−1^ at 1 C and 450–900 mA h g^−1^ at 2 C)^11^,^21^,^36^,^37^,^52^,^59^,^60^,^61^,^62^. Although some capacities reported at higher current densities (for example, 4 and 5 C) were relatively high (650–750 mA h g^−1^), the sulfur content of their composites was relatively low (40–65 wt%)^21^,^60^. The S@PGC (90% S) composite cathode exhibited high coulombic efficiencies (>98%) at all the current rates tested, confirming that the shuttling effect of the polysulphides was efficiently suppressed because of the C–S bonding between the sulfur nanoparticles and 3D PGC as well as the unique porous structure of the 3D S@PGC composite. The use of LiNO_3_ in the electrolyte also helped suppress polysulphide shuttling, improving the coulombic efficiency and enhancing the rate capability^63^,^64^, as demonstrated by experiments without LiNO_3_ in the electrolyte (Supplementary Fig. 14 and Supplementary Table 2). The relatively lower capacity retention at a lower charge/discharge rate was likely due to the incomplete oxidation of the insulating Li_2_S_2_ and Li_2_S (refs 55, 65) because the shuttling effect was efficiently suppressed and the coulombic efficiency was found to be higher than 98%.

The stability of the 3D S@PGC (90% S) composite cathode was evidenced by electrochemical impedance spectroscopy (EIS) before and after 200 cycles at 2 C (Fig. 6g). The corresponding Nyquist profiles were fitted to a widely used equivalent circuit (Supplementary Fig. 15)^66^. The electrolyte resistance (*R*_e_) and the charge-transfer resistance at the electrode/electrolyte interface (*R*_ct_) were determined to be 3.6 and 43.2 Ω before cycling and 4.6 and 47.9 Ω after cycling, respectively. The slight changes in *R*_e_ and *R*_ct_ after the charge/discharge cycles, which could be ascribed to the formation of a passive layer on the carbon frameworks^67^, suggest the high conductivity and good stability of the 3D S@PGC (90% S) cathode due to the stable 3D PGC framework and the C–S bonds between the sulfur nanoparticles and the 3D PGC framework. TEM observations also indicated the stability of the 3D S@PGC (90% S) composite electrode, as the porous structures of the 3D PGC remained intact and the sulfur nanoparticles were still firmly and homogeneously anchored to the PGC walls after 200 charge/discharge cycles at 2 C (Supplementary Fig. 16). In addition, the FTIR data of the electrode material indicated the retention of the C−S bonds after cycling (Supplementary Fig. 17), consistent with a previous study that reported the retention of C−S bonds after cycling^68^.

The rate capability of the 3D S@PGC (90% S) composite is depicted in Fig. 6h. The discharge capacity gradually decreased as the current rate increased from 0.2 to 5 C. At the maximum charge/discharge rate tested (5 C), the specific capacity remained high (500 mA h g^−1^) and the cycling remained stable; when the current rate was restored to 0.2 C, the composite recovered most of its capacity. Most importantly, the composite exhibited stable cycling performance over 1,000 charge/discharge cycles at 2 C (Fig. 6i). A high specific capacity (670 mA h g^−1^) was retained after 1,000 cycles. The calculated capacity retention is 61%, which corresponds to a very small capacity decay (0.039% per cycle). This finding demonstrates the excellent cycling stability of our Li–S batteries. Although other composites have been reported to exhibit a long cycling life when used as cathodes in Li–S batteries, the sulfur content employed in such materials was relatively low (ranging from 30 to 80%)^30^,^37^,^63^,^69^. Therefore, 3D S@PGC is a high-sulfur-content (up to 90%) cathode material that exhibits excellent cycling stability at a high current density. The pure sulfur cathode (the control) used under the same conditions exhibited a much lower specific capacity and worse cycling stability than those of the 3D S@PGC (90% S) composite (Supplementary Fig. 18).

## Discussion

The electrochemical performance of the 3D S@PGC (64% S) and 3D S@PGC (70% S) composites as cathodes in Li–S batteries were also evaluated. As summarized in Supplementary Fig. 19 and Supplementary Tables 3 and 4, 3D S@PGC composites with a lower sulfur content exhibited better performance: specifically, higher specific capacities, higher capacity retention and higher rate performance (Supplementary Note 3). These results are attributable to the smaller sizes of the sulfur nanoparticles in the 3D S@PGC composites with a relatively low sulfur content. Indeed, smaller sulfur nanoparticles have larger specific contact areas with the 3D PGC framework, which helps alleviate the shuttling effect and improves the cycle stability. Smaller particle sizes also facilitate electron and Li^+^ diffusion and lead to better sulfur utilization and a higher specific capacity. The larger specific surface areas effectively reduce the discharging current densities and the Li^+^ flux, thereby limiting the formation of a Li_2_S blocking layer at high charge/discharge rates^51^. Although the 3D S@PGC composites with a lower sulfur content exhibited higher specific capacities calculated on the basis of sulfur, the relatively low sulfur content reduced the overall volumetric capacity and energy density of the corresponding cathodes. Therefore, 3D S@PGC composites with relatively high sulfur content may be promising candidates for use in practical applications.

The excellent overall electrochemical performance of the 3D S@PGC composites can be attributed to the following factors that stem from the design of the materials. First, the *in situ* chemical deposition method allows access to composites with high sulfur content (up to 90 wt%) and affords the nanoscale distribution of the sulfur particles in the resultant 3D PGC network. As described above, nanosized sulfur particles facilitate a high sulfur utilization rate (82.5% for 3D S@PGC (90% S), 84.5% for 3D S@PGC (70% S) and 86% for 3D S@PGC (64% S) at 0.5 C). Second, the C–S bonds formed between the sulfur nanoparticles and 3D PGC can effectively prevent agglomeration of the sulfur nanoparticles, minimize the loss of lithium polysulphides to the electrolyte and suppress the shuttling effect during the charge/discharge cycles. Third, the 3D PGC networks that display high electrical conductivities, large surface areas and high mechanical flexibility confer high electrical conductivity and structural integrity to the electrodes. The numerous walls between the interconnected macropores may function as multilayered barriers to further mitigate the dissolution of polysulphides into the electrolyte. Finally, the unique interconnected hierarchical pores in the 3D PGC network facilitate access to the sulfur nanoparticles by the electrolyte and preserve the rapid transport of Li^+^ to the active material.

In conclusion, we report a new methodology that is facile and scalable and allows the *in situ* preparation of 3D S@PGC composites with a high sulfur content. The strategy utilizes Na_2_S as a sulfur precursor and NaCl and Na_2_S as a template for the porous structure of the resultant composite. The sulfur nanoparticles were homogenously distributed and covalently bonded to 3D PGC, as confirmed by various spectroscopic and microscopic techniques. Li–S batteries prepared using the composites as cathodes exhibited excellent performance; specifically, high sulfur utilization, high specific capacities, good cycling stabilities and high rate capabilities were observed. Notably, Li–S batteries prepared using 3D S@PGC (90% S) as a cathode displayed a long cycling stability, with a capacity decay of only 0.039% per cycle over 1,000 cycles at a high charge/discharge current (2 C). Overall, the methodology described herein offers a new avenue for the fabrication of cathode materials based on carbon–sulfur hybridized nanostructures for use in high-performance Li–S batteries. We believe that the strategy may also inspire the preparation of other 3D porous structures for use in other areas, including applications in catalysis, selective adsorption, separations and sensing.

## Methods

### Materials

All reagents were purchased from commercial sources and used without further purification. All solvents used were purified using standard procedures.

### Representative synthesis of 3D S@PGC composites

In a typical synthesis, Na_2_S·9H_2_O (2.0 g), NaCl (5.0 g) and glucose (0.8 g) were dissolved in DI water (15 ml). The resultant solution was frozen in liquid nitrogen, and the water in the mixture was removed via freeze-drying. The resultant gel was ground into a fine powder and then heated at 750 °C for 2 h under an atmosphere of argon. A black powder was obtained and subsequently stirred in an aqueous solution of Fe(NO_3_)_3_ (20 g Fe(NO_3_)_3_·9H_2_O in 150 ml DI water) for 40 h to dissolve the residual NaCl crystals and to deposit the sulfur. Afterwards, the black powder product was washed several times with DI water and centrifuged to afford the desired composite. Composites with various sulfur contents were synthesized by using different amounts of glucose (0.9 and 1.0 g) in the aforementioned procedure.

### Characterization

X-ray diffraction data were collected on a Bruker D8 Focus diffractometer using an incident wavelength of 0.154 nm (Cu Kα radiation) and a Lynx-Eye detector. Raman spectra were recorded on a Renishaw inVia-Reflex confocal Raman microscope at an excitation wavelength of 532 nm. TGA measurements were carried out using a TGA Q50 at a scanning rate of 10 °C min^−1^. SEM observations were performed on a field-emission SEM (Hitachi S-4800) equipped with EDS. TEM images were obtained using a JEOL-2100F microscope operated under an accelerating voltage of 200 kV. EDS analysis was also performed on Tecnai F20 scanning transmission electron microscope operated at 200 keV using an Oxford detector with a beam current of ∼1 nA. N_2_ adsorption–desorption isotherms and pore-size distribution were obtained at 77 K using a QuadraSorb SI MP apparatus. The total specific surface areas of the samples were calculated via the Brunauer–Emmett–Teller method. The pore-size distribution was calculated via the density functional theory model. XPS spectra were recorded on a PHI Quantera Scanning X-ray Microprobe using monochromated Al Kα radiation (1486.7 eV). FTIR spectra were recorded on an Excalibur 3100 spectrometer with a resolution of 0.2 cm^−1^ using KBr pellets.

### Electrochemistry

The 3D S@PGC composites were combined with conductive carbon and poly(vinylidene fluoride) as a binder with a mass ratio of 80:10:10 and milled into a slurry with N-methylpyrrolidone. The slurry was then blade cast onto a carbon-coated Al foil and dried at 50 °C for 10 h in a vacuum oven. The loading density of sulfur was ca. 2.36 mg cm^−2^. CR2032 coin cells were assembled in an argon-filled glove box employing the 3D S@PGC-coated Al foil as the cathode, a porous membrane (Celgard 3501) as the separator, and lithium foil as the reference/counter electrode. The electrolyte used was lithium bis(trifluoromethane)sulphonimide (0.38 M) and lithium nitrate (0.31 M) in a solvent mixture of 1,3-dioxolane and 1,2-dimethoxy ethane (1:1 v/v). Pristine sulfur electrodes were fabricated under similar conditions. Cyclic voltammetry curves were collected using a CHI 660E electrochemical workstation at a scan rate of 0.1 mV s^−1^ from 3.0 to 1.5 V. Cycling tests of the batteries were galvanostatically performed at various charge/discharge rates within a potential window of 1.5–3.0 V versus Li^+^/Li. The electrochemical impedance spectroscopy data were recorded using a Zennium 40088 electrochemical workstation by applying a sine wave with an amplitude of 10 mV over a frequency range of 100 kHz to 10 mHz.

## Additional information

**How to cite this article:** Li, G. *et al.* Three-dimensional porous carbon composites containing high sulfur nanoparticle content for high-performance lithium–sulfur batteries. *Nat. Commun.* 7:10601 doi: 10.1038/ncomms10601 (2016).

## Supplementary Material

Supplementary Information Supplementary Figures 1-19, Supplementary Tables 1-4, Supplementary Notes 1-3 and Supplementary References
