# Supplementary material for: Three-dimensional porous carbon composites containing high sulfur nanoparticle content for high-performance lithium–sulfur batteries
Source: Nat Commun. 2016 Feb 1;7:10601. doi: 10.1038/ncomms10601 (PMC4740444; doi:10.1038/ncomms10601)
Supplement: Supplementary Information — Supplementary Figures 1-19, Supplementary Tables 1-4, Supplementary Notes 1-3 and Supplementary References [file ncomms10601-s1.pdf]

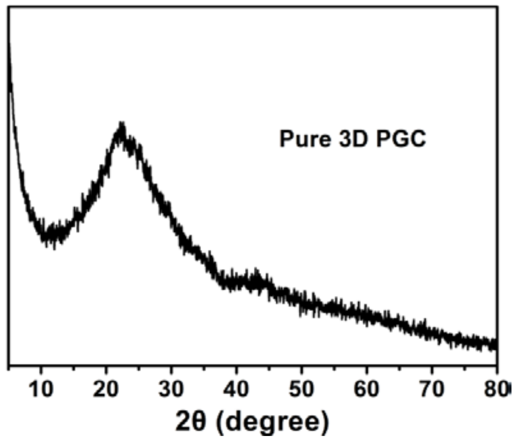

Supplementary Figure 1 XRD pattern of pure 3D PGC framework. The pure 3D PGC was obtained by immersing NaCl–Na_2S@GC in water to remove the NaCl and Na_2S. The broad reflection peak in the range of 15–35° was attributed to the PGC.

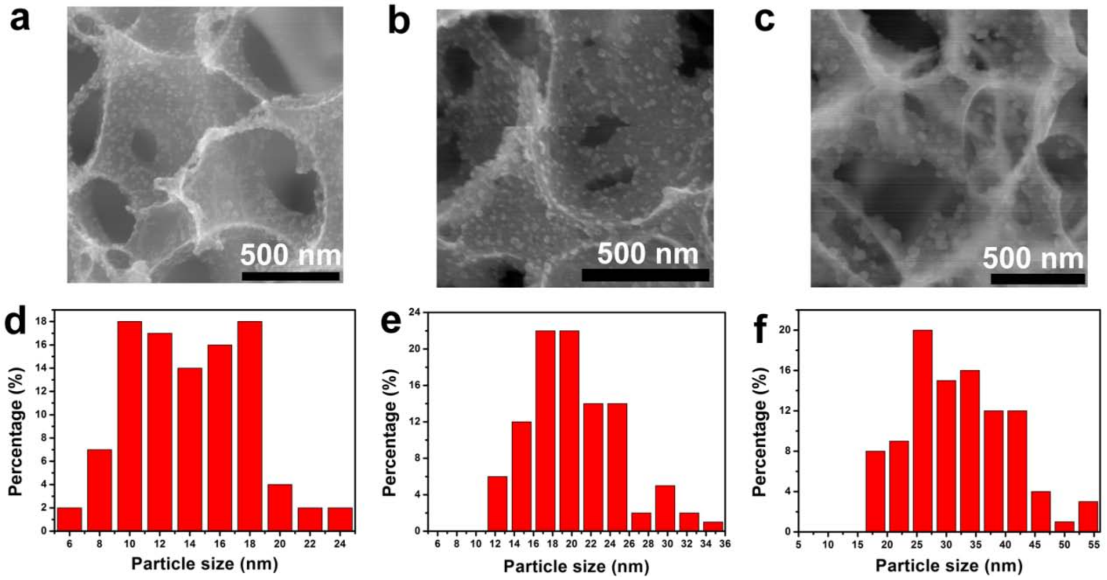

Supplementary Figure 2 Size analysis of the sulphur nanoparticles in the 3D S@PGC composites. (**a**–**c**) SEM images of (**a**) 3D S@PGC (64% S), (**b**) 3D S@PGC (70% S) and (**c**) 3D S@PGC (90% S) composites, showing that sulphur nanoparticles were attached to the PGC walls. (**d–f**) Size statistics of the sulphur nanoparticles (based on 100 particles), indicating that the nanoparticles featured size distributions in range of (**d**) 6–24 nm for 3D S@PGC (64% S) composite, (**e**) 12–35 nm for 3D S@PGC (70% S) composite, and (**f**) 18–54 nm for 3D S@PGC (90% S) composite, respectively.

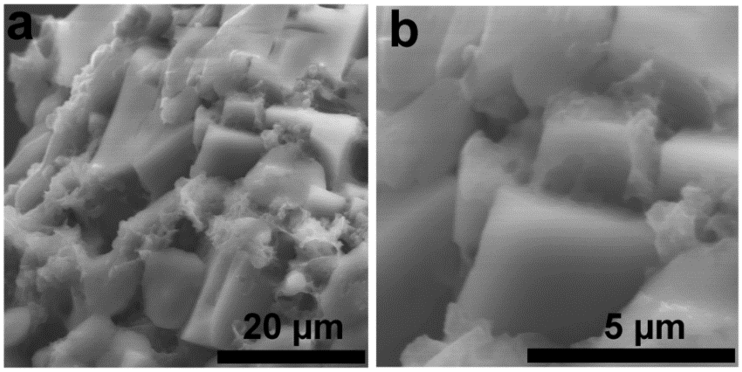

Supplementary Figure 3 SEM images of the 3D NaCl–Na_2S@GC. (**a**) At a low magnification and (**b**) at a high magnification. These images show the 3D self-stacking of the NaCl and Na_2S crystals and that the crystals were wrapped with a layer of GC.

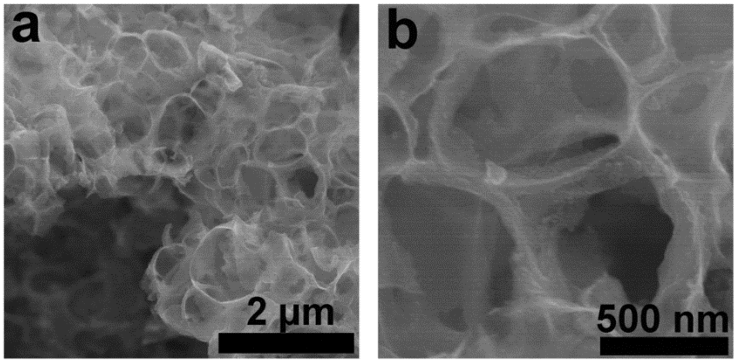

Supplementary Figure 4 SEM images of pure 3D PGC. (**a**) At a low magnification and (**b**) at a high magnification. The pure 3D PGC were obtained by immersing the NaCl–Na_2S@GC in water to remove the NaCl and Na_2S. The SEM images indicated that the 3D PGC displayed a unique network comprised of interconnected macropores.

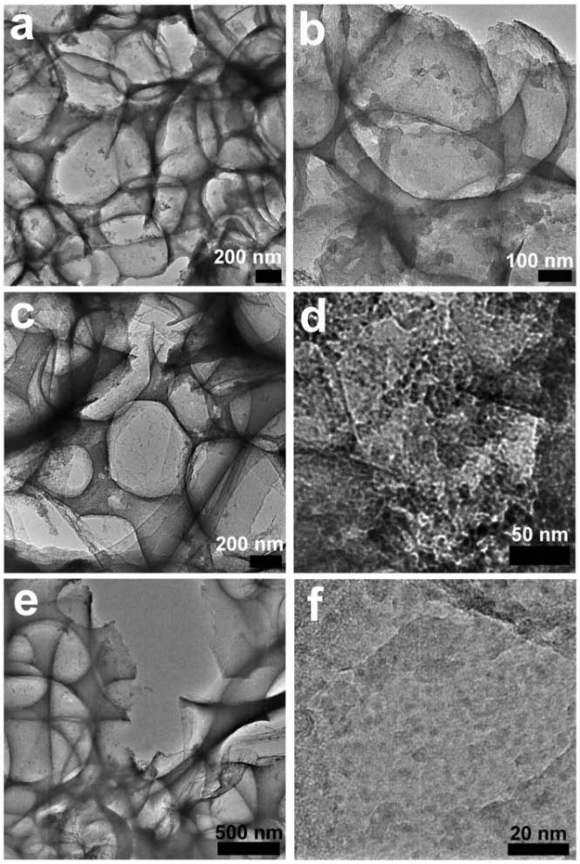

Supplementary Figure 5 Morphological characterization of the 3D S@PGC composites after being subjected to an elongated period of sonication during TEM sample preparation. (**a**, **c**, **e**) TEM images of (**a**) 3D S@PGC (90% S) composite, (**c**) 3D S@PGC (70% S) composite, and (**e**) 3D S@PGC (64% S) composite. The images revealed that the 3D structures remained intact. (**b**, **d**, **f**) TEM images of (**b**) 3D S@PGC (90% S) composite, (**d**) 3D S@PGC (70% S) composite, and (**f**) 3D S@PGC (64% S) composite at a higher magnification, showing that sulphur nanoparticles were homogeneously attached to the PGC walls. These data indicate the high mechanical flexibility of the 3D PGC network and the robust attachment of the sulphur nanoparticles to the PGC walls.

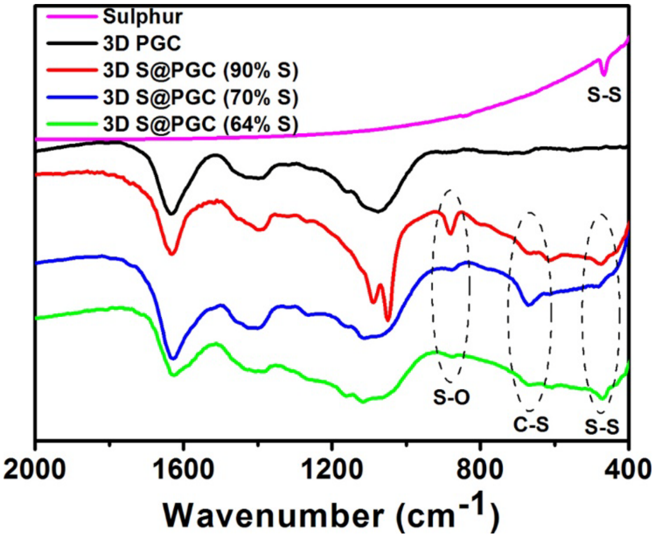

Supplementary Figure 6 FT-IR spectra of sulphur, pure 3D PGC and the 3D S@PGC composites. The pure 3D PGC was obtained by immersing the NaCl–Na_2S@GC in water to remove the NaCl and Na_2S. In the FT-IR spectra of the 3D S@PGC composites, the vibration peak at ca. 880 cm^{-1} was ascribed to S–O bonds[1], while the vibration peaks at ca. 671 and 474 cm^{-1} were assigned to C–S and S–S bonds, respectively[2,3].

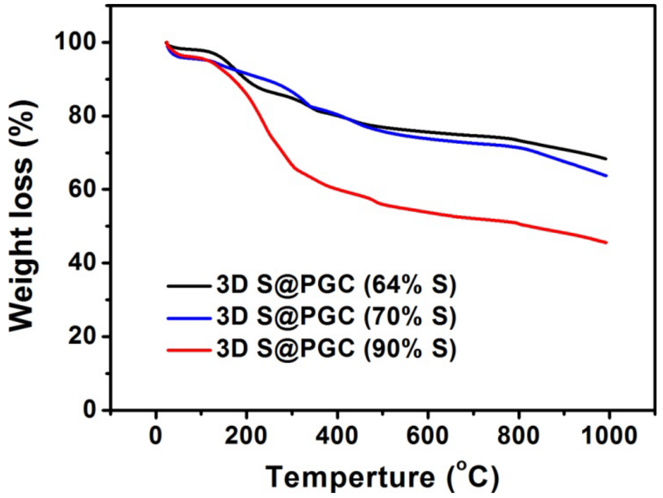

Supplementary Figure 7 TGA curves of Soxhlet extracted 3D S@PGC composites. The Soxhlet extraction was performed using CS_2 for 48 h. The samples did not show obvious weight losses corresponding to evaporation of pure sulphur, implying that the sulphur was bonded to PGC matrix[4]. The sulphur contents of the Soxhlet extracted samples were roughly determined to be ca. 48, 27, and 25 wt% according to the weight losses measured at 700 °C.

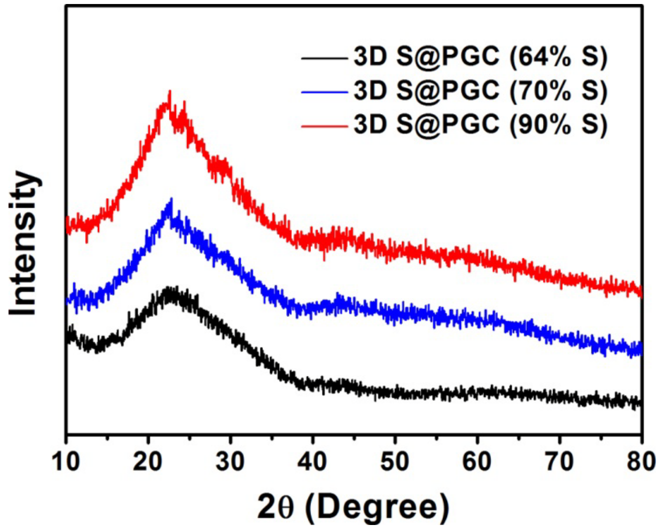

Supplementary Figure 8 XRD patterns of Soxhlet extracted 3D S@PGC composites. The Soxhlet extracted samples did not show Bragg reflections of pure sulphur, implying that the sulphur was bonded to PGC matrix[5].

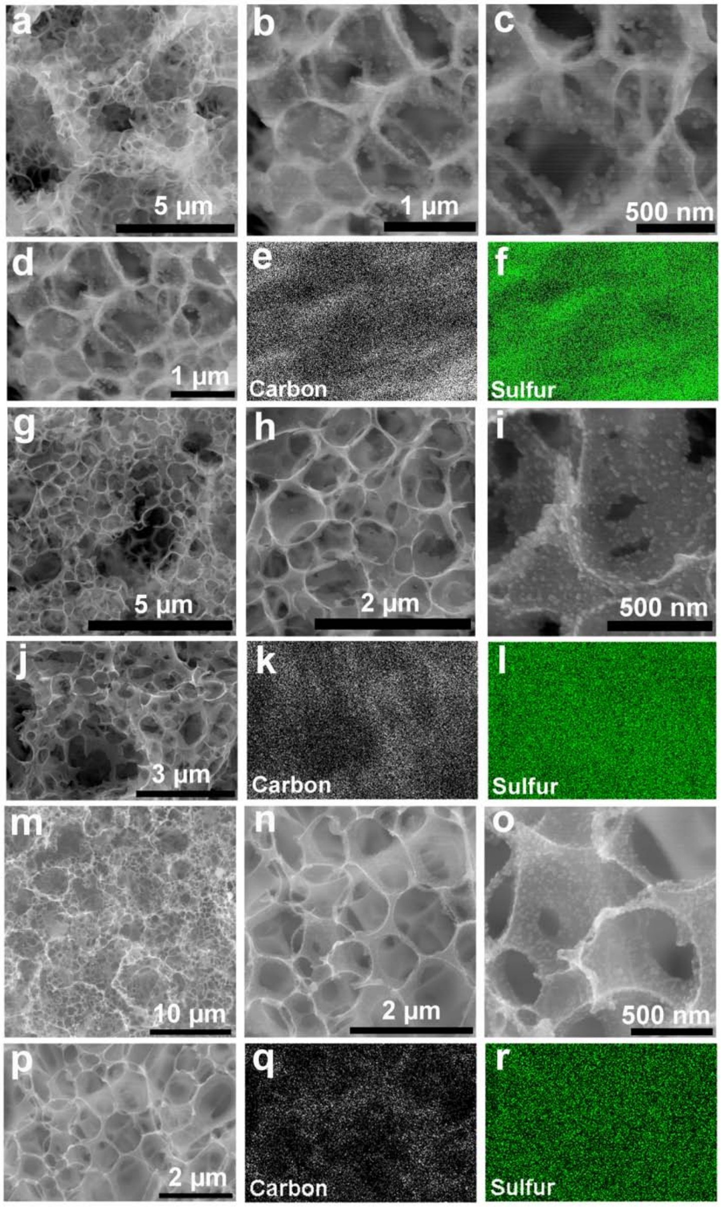

Supplementary Figure 9 SEM characterization and elemental analysis of the 3D S@PGC composites. (**a–c**) SEM images of the 3D S@PGC (90% S) composite at different magnifications. (**d**) An SEM image of the 3D S@PGC (90% S) composite. (**e**, **f**) EDS elemental maps of (**e**) carbon and (**f**) sulphur, which were collected from the entire area shown in **d**. (**g–i**) SEM images of the 3D S@PGC (70% S) composite at different magnifications. (**j**) An SEM image of the 3D S@PGC (70% S) composite. (**k**, **l**) EDS elemental maps of (**k**) carbon and (**l**) sulphur, which were collected from the entire area shown in **j**. (**m–o**) SEM images of the 3D S@PGC (64% S) composite at different magnifications. (**p**) An SEM image of the 3D S@PGC (64% S) composite. (**q**, **r**) EDS elemental maps of (**q**) carbon and (**r**) sulphur, which were collected from the entire area shown in **p**.

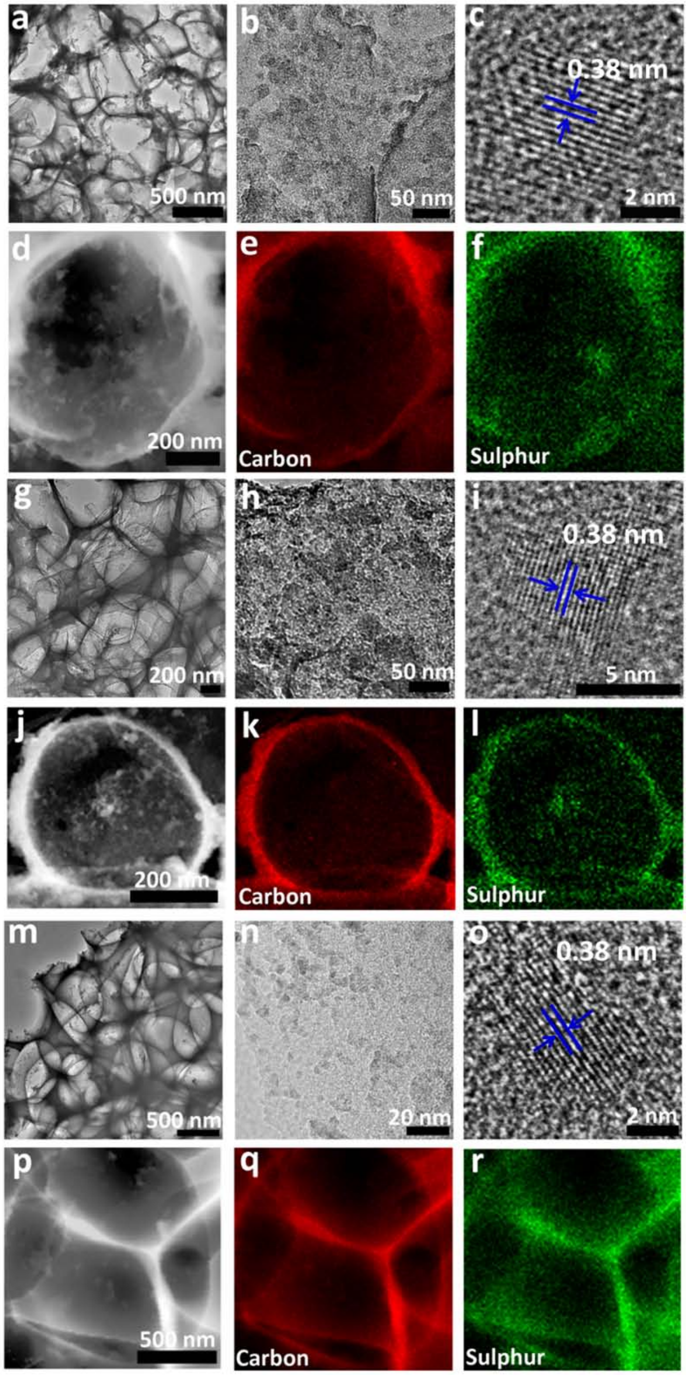

Supplementary Figure 10 TEM characterization and elemental analysis of the 3D S@PGC composites. (**a–c**) TEM images of the 3D S@PGC (90% S) composite at different magnifications. (**d**) A TEM image of the 3D S@PGC (90% S) composite. (**e**, **f**) EDS elemental maps of (**e**) carbon and (**f**) sulphur, which were collected from the entire area shown in **d**. (**g–i**) TEM images of the 3D S@PGC (70% S) composite at different magnifications. (**j**) A TEM image of the 3D S@PGC (70% S) composite. (**k**, **l**)

EDS elemental maps of (**k**) carbon and (**l**) sulphur, which were collected from the entire area shown in **j**. (**m–o**) TEM images of the 3D S@PGC (64% S) composite at different magnifications. (**p**) A TEM image of the 3D S@PGC (64% S) composite. (**q**, **r**) EDS elemental maps of (**q**) carbon and (**r**) sulphur, which were collected from the entire area shown in **p**. These data indicate that the unique honeycomb-like network in the composites are maintained in the composites, and the the sulphur particles remained nanosized in scale.

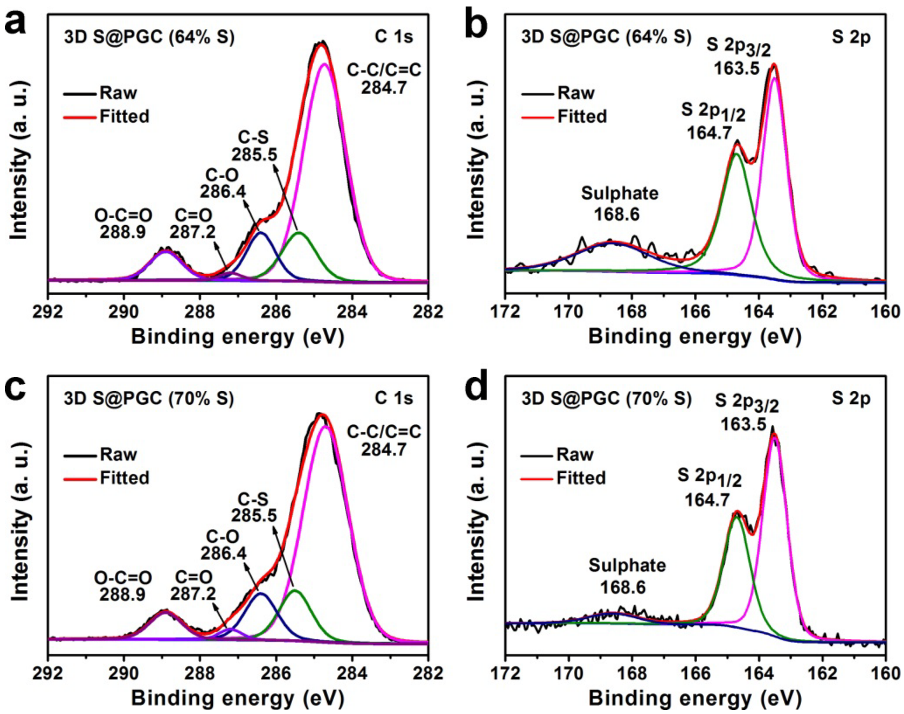

Supplementary Figure 11 XPS analysis of the 3D S@PGC (64% S) and 3D S@PGC (70% S) composites. (**a**) C 1*s* XPS and (**b**) S 2*p* XPS spectra of the 3D S@PGC (64% S) composite. (**c**) C 1*s* XPS and (**d**) S 2*p* XPS spectra of the 3D S@PGC (70% S) composite.

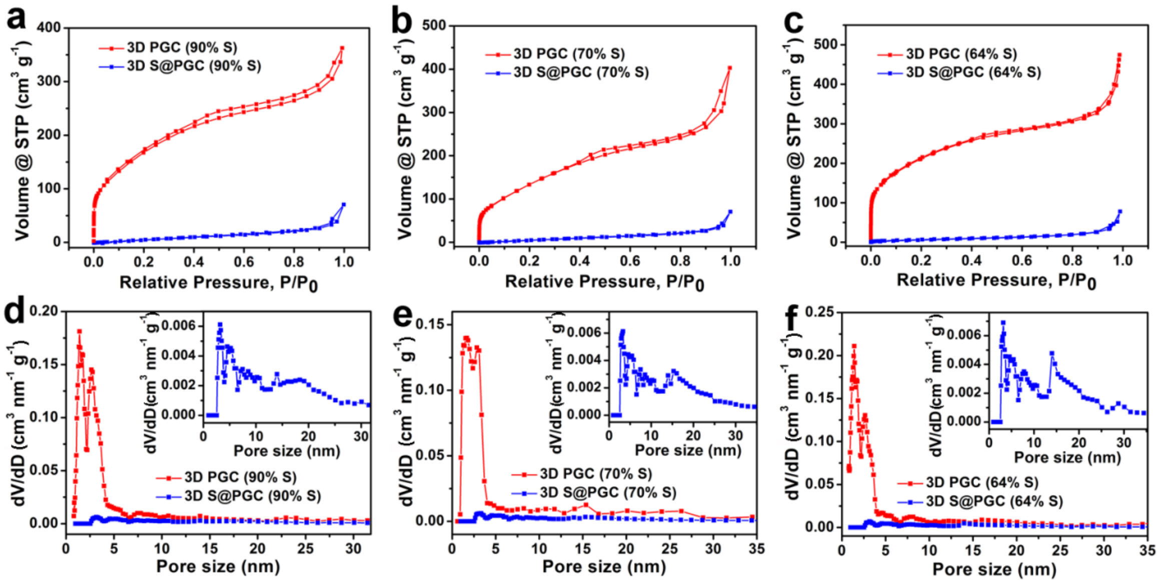

Supplementary Figure 12 N_2 adsorption/desorption analysis of the 3D S@PGC composites and the corresponding 3D PGC frameworks. (**a–c**) N_2 adsorption/desorption isotherms of (**a**) the 3D S@PGC (90% S) composite and corresponding 3D PGC, (**b**) the 3D S@PGC (70% S) composite and corresponding 3D PGC, and (**c**) the 3D S@PGC (64% S) composite and corresponding 3D PGC. (**d–f**) Pore size distributions of (**d**) the 3D S@PGC (90% S) composite and corresponding 3D PGC, (**e**) the 3D S@PGC (70% S) composite and corresponding 3D PGC, and (**f**) the 3D S@PGC (64% S) composite and corresponding 3D PGC. The insets shown in **d–f** are the magnified graphs of the 3D S@PGC composites.

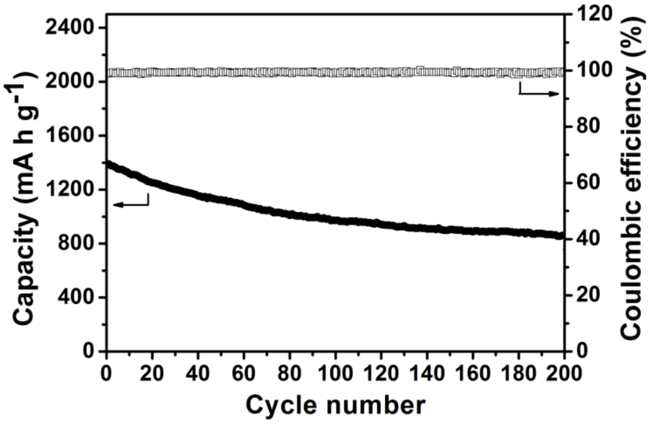

Supplementary Figure 13 Cycling performance of the 3D S@PGC (90% S) composite cathode at a charge/discharge rate of 0.5 C. An initial specific capacity was measured to be as high as 1382 mA h g^{-1}, corresponding to 82.5% utilization of sulphur based on the theoretical value (1675 mA h g^{-1}).

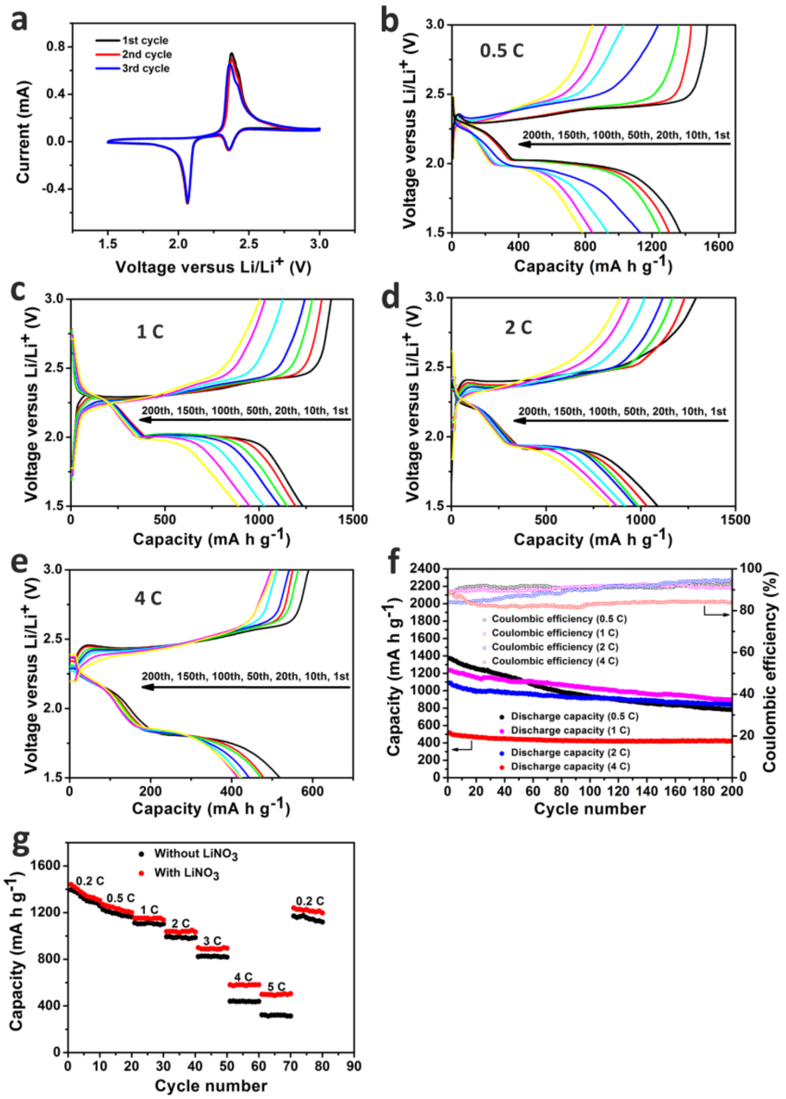

Supplementary Figure 14 Electrochemical performance of the 3D S@PGC (90% S) composite as cathode material for the Li–S batteries fabricated without $LiNO_3$ in electrolyte. Electrolyte: lithium bis(trifluoromethane)sulfonimide (1 M) in a mixture solvent of 1,3-dioxolane and 1,2-dimethoxy ethane (1:1 v/v). (**a**) CV profiles. (**b–e**) Charge/discharge curves measured at charge/discharge rates of (**b**) 0.5 C, (**c**) 1 C, (**d**) 2 C, and (**e**) 4 C. (**f**) Cycling performance measured at 0.5, 1, 2, and 4 C. (**g**) Rate performances measured for cells fabricated with/without $LiNO_3$ in electrolyte for comparison.

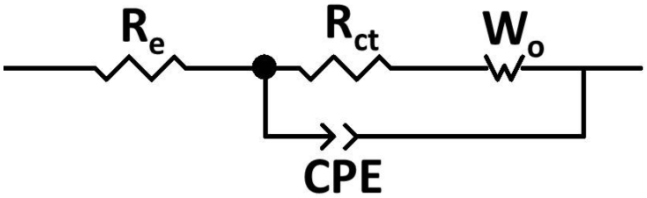

Supplementary Figure 15 The equivalent circuit used for fitting the EIS curves. R_e refers to the resistance of electrolyte, R_{ct} refers to the charge transfer resistance at the electrode/electrolyte interface, W_o refers to the Warburg impedance, and CPE refers to the constant phase element[5].

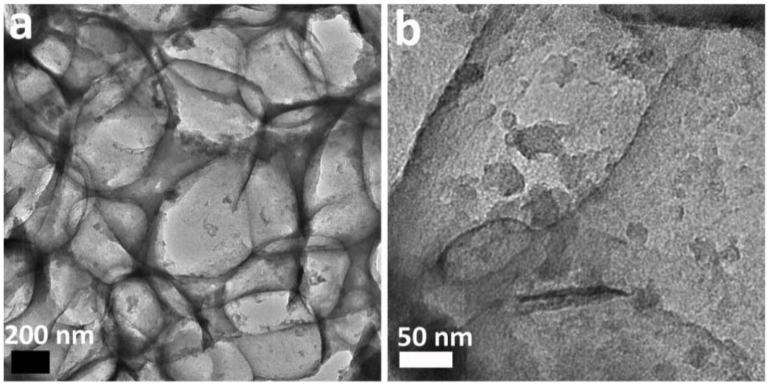

Supplementary Figure 16 TEM images of the 3D S@PGC (90% S) composite as cathode for a Li–S battery after 200 charge/discharge cycles at a rate of 2 C. (**a**) At a low magnification and (**b**) at a high magnification. These data indicated that the structure of the 3D S@PGC remained intact after the charge/discharge cycling, and that the sulphur nanoparticles still anchored to the PGC walls firmly and homogeneously, which could be contributed to the C–S bonds between the sulphur nanoparticles and the PGC.

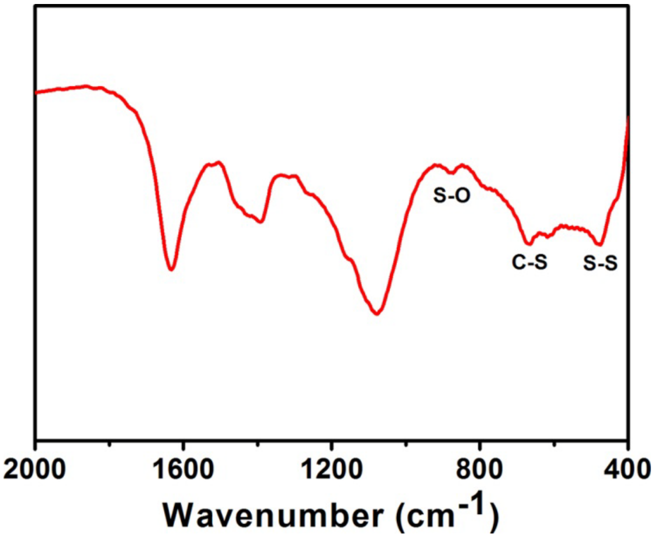

Supplementary Figure 17 FT-IR spectrum of the 3D S@PGC (90% S) cathode material after charge/discharge cycles. To collect the FT-IR spectrum, batteries were opened at a charged state. Then the cathode material was scraped off from the Al foil, washed with NMP, and fully dried. Detection of the characteristic vibration peak at 671 cm^{-1} indicated the retention of C-S bonds, which was in agreement with previous report[6].

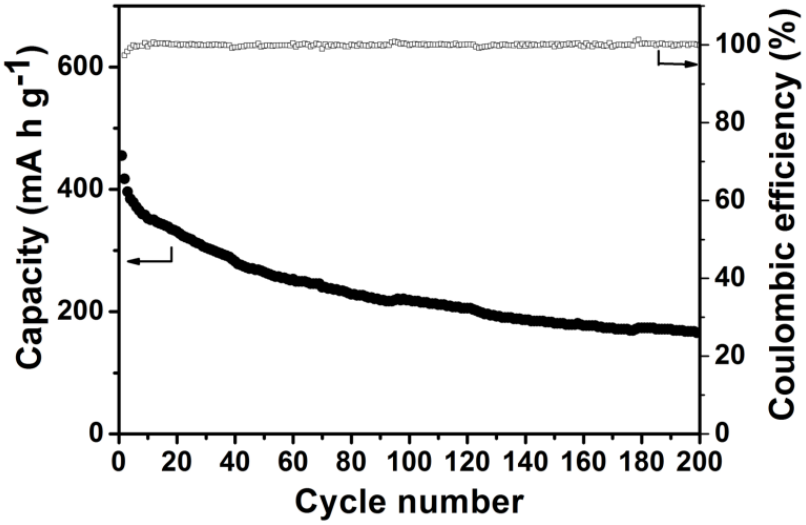

Supplementary Figure 18 Cycling performance of a pure sulphur cathode for Li–S batteries measured at charge/discharge rate of 1 C. An initial specific capacity was measured to be 450 mA h g^{-1}. After 200 cycles, the capacity retention was calculated to be 30%.

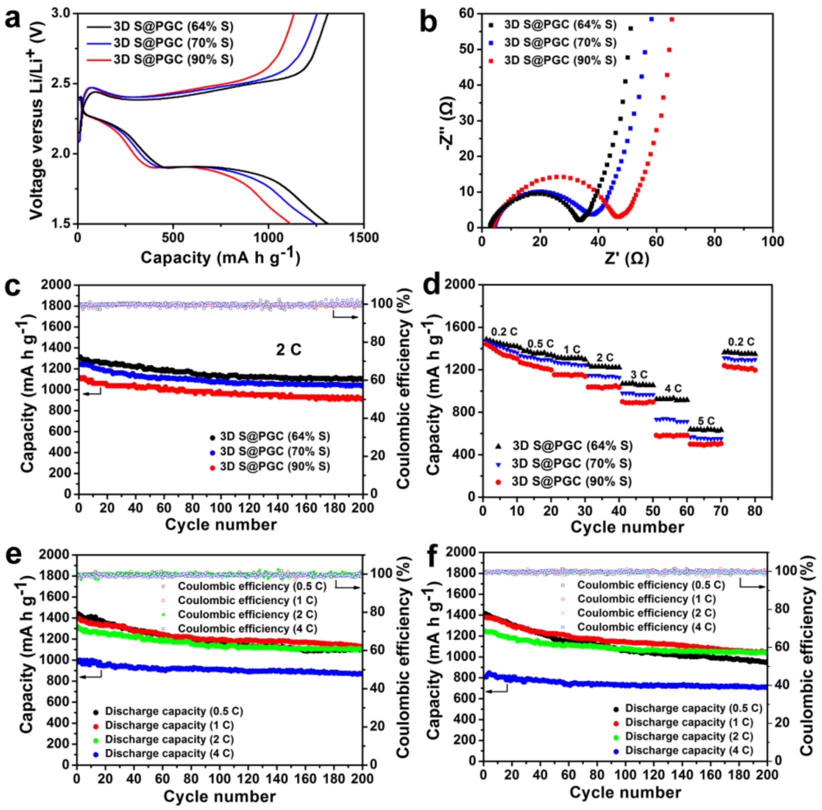

Supplementary Figure 19 Electrochemical performances of the 3D S@PGC composites with different contents of sulphur as cathode for Li–S batteries. (**a**) Initial galvanostatic discharge/charge profiles at 2 C. (**b**) EIS curves of the as-prepared batteries. (**c**) Cycling performance and coulombic efficiency at 2 C. (**d**) Rate performance at current densities from 0.2 to 5 C. (**e**) Cycling performance of the 3D S@PGC (64% S) composite as cathode material at charge/discharge rates of 0.5, 1, 2, and 4 C. (**f**) Cycling performance of the 3D S@PGC (70% S) composite as cathode material at charge/discharge rates of 0.5, 1, 2, and 4 C.

Supplementary Table 1 Summarization of 3D S@PGC composites containing different contents of sulphur.

Entry	Starting materials				Content of sulphur (bonded S + unbounded S) (wt%)[a]	Size distribution of sulphur nanoparticles (nm)[b]
	$Na_2S{\cdot}9H_2O$ (g)	Glucose (g)	NaCl (g)	H_2O (mL)		
Sample 1	2	1.0	5	15	64% (12% + 52%)	6–24
Sample 2	2	0.9	5	15	70% (11% + 59%)	12–35
Sample 3	2	0.8	5	15	90% (9% + 81%)	18–54

[a]The contents of bonded and unbounded sulphur in 3D S@PGC composites were calculated by comparing the sulphur content in the as-prepared 3D S@PGC composites to that in the Soxhlet extracted samples.

[b]The size distributions of the sulphur nanoparticles were calculated from the SEM images shown in Supplementary Fig. 2.

Supplementary Table 2. Comparison of the specific capacities before and after 200 cycles and the capacity retention at different charge/discharge rates for the 3D S@PGC (90% S) composite as cathode measured with/without $LiNO_3$ in electrolyte.

Rate	Without $LiNO_3$ in electrolyte			With $LiNO_3$ in electrolyte		
	Specific capacity (mA h g^{-1})		Capacity retention	Specific capacity (mA h g^{-1})		Capacity retention
	1st cycle	200th cycle		1st cycle	200th cycle	
0.5 C	1373	782	57%	1382	859	62%
1 C	1236	890	72%	1242	917	74%
2 C	1090	841	77%	1115	920	83%
4 C	518	422	81%	638	548	86%

Supplementary Table 3 R_e and R_{ct} values of the 3D S@PGC composites as cathodes for Li–S batteries.

	3D S@PGC (64% S)	3D S@PGC (70% S)	3D S@PGC (90% S)
R_e (Ω)	3.3	3.7	3.6
R_{ct} (Ω)	30.7	33.8	43.2

Supplementary Table 4 Summarization of the electrochemical performances of the 3D S@PGC composites as cathodes for Li–S batteries.

Rate	3D S@PGC (90% S)			3D S@PGC (70% S)			3D S@PGC (64% S)		
	Capacity (mA h g^{-1})		Capacity retention	Capacity (mA h g^{-1})		Capacity retention	Capacity (mA h g^{-1})		Capacity retention
	1st cycle	200th cycle		1st cycle	200th cycle		1st cycle	200th cycle	
0.5 C	1382	859	62%	1415	955	67.5%	1440	1103	76.6%
1 C	1242	917	74%	1378	1045	75.8%	1397	1129	80.8%
2 C	1115	920	83%	1247	1042	83.6%	1314	1109	84.4%
4 C	638	548	86%	807	704	87.2%	981	866	88.3%

Supplementary Note 1.

Plausible mechanism for the formation of C–S bonds in the composites. The oxidation of Na_2S by $Fe(NO_3)_3$ results in the formation of the free radical $HS^{\bullet}$, which is then transformed to the radical anion $S^{\bullet-}$ and the disulfide ion S_2^{2-}; subsequent oxidation affords radical anions ($S_3^{\bullet-}$, $S_4^{\bullet-}$, $S_6^{\bullet-}$, etc.), and negative-charged polysulfides (S_3^{2-}, S_4^{2-}, S_6^{2-}, etc.)[9]. These reactive intermediate species may attack the C=C bonds or the oxygen-containing functional groups of the PGC, and ultimately result in the formation of C–S bonds.

Supplementary Note 2.

N_2 adsorption/desorption analysis. The 3D PGC frameworks of the 3D S@PGC composites were obtained by immersing the NaCl–Na_2S@GC in water to remove the NaCl and Na_2S. A series of N_2 adsorption/desorption analyses of the 3D S@PGC composites as well as the corresponding 3D PGC indicated that all the 3D S@PGC composites have smaller specific surface areas than the corresponding 3D PGC frameworks: 38.8 vs. 628.5 $m^2 g^{-1}$ for the 3D S@PGC (90% S) composite and corresponding 3D PGC, 22.5 vs. 529.0 $m^2 g^{-1}$ for the 3D S@PGC (70% S) composite and corresponding 3D PGC, and 27.8 vs. 754.9 $m^2 g^{-1}$ for the 3D S@PGC (64% S) composite and corresponding 3D PGC. Likewise, the 3D S@PGC composites also have smaller total pore volumes than the corresponding 3D PGC frameworks: 0.11 vs. 0.56 $cm^3 g^{-1}$ for the 3D S@PGC (90% S) composite and corresponding 3D PGC, 0.10 vs. 0.62 $cm^3 g^{-1}$ for the 3D S@PGC (70% S) composite and corresponding 3D PGC, and 0.12 vs. 0.74 $cm^3 g^{-1}$ for the 3D S@PGC (64% S) composite and corresponding 3D PGC. The marked decreases in specific surface area and total pore volume, upon deposition of the sulphur nanoparticles, indicated that sulphur occupied the volumes of the pores in the 3D PGC frameworks. Comparison of the pore size distributions of the 3D S@PGC composites and the corresponding 3D PGC frameworks indicated that the latter contained plenty of micro- and mesopores with size ranging from ca. 1.5 to 25 nm, while they were almost filled with sulphur in the composites.

Supplementary Note 3.

The 3D S@PGC composites with different sulphur contents for Li–S battery cathode materials. Supplementary Fig. 19a presents the initial galvanostatic discharge/charge profiles of the 3D S@PGC

composites as cathodes at 2 C. All discharge curves show two plateaus, corresponding to the two-stage reduction reactions of elemental sulphur to lithium polysulphides ($Li_2S_{4–8}$), and then to Li_2S_2/Li_2S, respectively. The 3D S@PGC (64% S) composite cathode exhibited larger overall specific capacity than those prepared using the composites containing higher contents of sulphur. This can be contributed to the higher utilization rate of the sulphur nanoparticles contained in the 3D S@PGC (64% S) due to their smaller sizes. All the cathodes prepared using the 3D S@PGC composites with different contents of sulphur show EIS curves that comprise a depressed semicircle in the high frequency region and an inclined line in the low frequency region (Supplementary Fig. 19b). The R_e and R_{ct} were estimated using the equivalent circuit shown in Supplementary Fig. 15 and summarized in Supplementary Table 3. The R_{ct} underwent a gradual decline as the content of sulphur in the composites decreased, indicating faster electrode kinetics. Since the 3D S@PGC composites with relatively lower contents of sulphur exhibited relatively smaller sizes of the sulphur nanoparticles (Supplementary Table 1), the faster electrode kinetics could be contributed to the smaller sulphur nanoparticles[7]. Smaller sulphur nanoparticles resulted in shorter pathway for the transport of ions as well as larger specific contact area and improved electrical contact between the PGC framework and the sulphur nanoparticles[8]. While all the 3D S@PGC composites as cathodes show good cycling stabilities at charge/discharge rate of 2 C (Supplementary Fig. 19c), the 3D S@PGC composites with relatively lower contents of sulphur exhibited relatively better rate performances (Supplementary Fig. 19d). Finally, the cycling stabilities of the 3D S@PGC (64% S) and the 3D S@PGC (70% S) as cathodes were evaluated at different charge/discharge rates (i.e., 0.5, 1, 2, and 4 C). As summarized in Supplementary Table 4, the 3D S@PGC composites with relatively lower contents of sulphur exhibited relatively better cycling stabilities, which were also contributed to the relatively smaller sulphur nanoparticles that could be more effectively immobilized to the PGC framework due to the relatively larger specific contact areas with the PGC walls.

On the other hand, although the 3D S@PGC composites with relatively lower contents of sulphur exhibit better specific capacities on the basis of sulphur, the low sulphur contents in composites mean low sulphur contents in cathodes, which will greatly reduce the overall volumetric capacities and energy densities of the cathodes. Therefore, the 3D S@PGC composites containing relatively higher contents of sulphur, with comparable electrochemical performances, are the more promising candidates for practical applications.

Supplementary References:

1. Wang, X., Gao, Y., Wang, J., Wang, Z., Chen, L. Chemical adsorption: Another way to anchor polysulfides. *Nano Energy* **12**, 810–815 (2015).
2. Wang, X., Wang, Z., Chen, L. Reduced graphene oxide film as a shuttle-inhibiting interlayer in a lithium-sulfur battery. *J. Power Sources* **242**, 65–69 (2013).
3. Yu, X. U., *et al.* Stable-cycle and high-capacity conductive sulfur-containing cathode materials for rechargeable lithium batteries. *J. Power Sources* **146**, 335–339 (2005).
4. Zhang, Y. Z., Liu, S., Li, G. C., Li, G. R., Gao, X. P. Sulfur/polyacrylonitrile/carbon multi-composites as cathode materials for lithium/sulfur battery in the concentrated electrolyte. *J. Mater. Chem. A* **2**, 4652–4659 (2014).
5. Narayanan, S. R., Shen, D. H., Surampudi, S., Attia, A. I., Halpert, G. Electrochemical impedance spectroscopy of lithium-titanium disulfide rechargeable cells. *J. Electrochem. Soc.* **140**, 1854–1861 (1993).
6. Fanous, J., Wegner, M., Grimminger, J., Andresen, A., Buchmeiser, M. R. Structure-related electrochemistry of sulfur-poly(acrylonitrile) composite cathode materials for rechargeable lithium batteries. *Chem. Mater.* **23**, 5024–5028 (2011).
7. Li, W., *et al.* Understanding the role of different conductive polymers in improving the nanostructured sulfur cathode performance. *Nano Lett.* **13**, 5534–5540 (2013).
8. Chen, H. W., *et al.* Monodispersed sulfur nanoparticles for lithium sulfur batteries with theoretical performance. *Nano Lett.* **15**, 798–802 (2015).
9. Steudel, R. Mechanism for the formation of elemental sulfur from aqueous sulfide in chemical and microbiological desulfurization processes. *Ind. Eng. Chem. Res.* **35**, 1417−1423 (1996).
